# Can we predict the IVF/ICSI live birth rate?

**DOI:** 10.5935/1518-0557.20190043

**Published:** 2019

**Authors:** José Luis Metello, Claudia Tomás, Pedro Ferreira

**Affiliations:** 1 Division of Reproductive Endocrinology and Infertility, Garcia de Orta Hospital, Almada, Portugal

**Keywords:** predictor, live-birth rate, *in vitro* fertilization, antimullerian hormone, antral follicle count, age

## Abstract

**Objectives:**

To find a pretreatment predictor for achieving a live birth. Assisted reproduction technology with IVF/ICSI is the ultimate chance for some couples to conceive a child. The expectations are high and it is important to give them a realistic perspective about the chances of achieving a live birth.

**Methods:**

A retrospective cohort study of all IVF/ICSI cycles performed in our center between 2012 and 2016. We considered only those cycles with a live birth delivery after 24 weeks, or cycles with no surplus embryos left. The following data was evaluated: AMH; AFC; age; BMI; previous diagnosis; type of treatment; number of previous deliveries; ethnicity, smoking status. Univariate and multivariate analysis were used to examine the association of live birth with baseline patient characteristics. We determined the odds-ratio for all the statistically significant variables (*p*<0.05), in a multivariate model. The results are presented according to the predictors founded.

**Results:**

739 cycles were evaluated: 9.1% were canceled; 10.2% did not have oocytes; 15.6% did not have D2 embryos; 31.4% achieved a live birth. The univariate analysis revealed statistically significant differences regarding AMH, AFC and women’s age between couples with and without a live birth (*p*<0.001), and the cause of infertility. We found no association with live births in other variables. These variables were categorized and used in a multivariate analysis.

**Conclusion:**

Age, AMH, AFC and cause, when sub-classified, are independently associated with the results of an IVF/ICSI treatment. These results enable couples to face real expectations in their particular scenario.

## INTRODUCTION

Infertility is defined as the failure to conceive within 12 months of regular unprotected intercourse, affects approximately one in six couples and many of those with prolonged unresolved infertility will be treated with Assisted Reproduction Treatments (ART) regardless of the cause (Leijdekkers *et al*., 2018). The increase in IVF/ICSI cycles is not caused by a sudden epidemic of infertility, but by increased access and by an expansion of their indications (van Loendersloot *et al*., 2014). Unfortunately, doing an IVF/ICSI cycle is not a guarantee of success. Some reports refer that up to 38-49% of couples that start IVF will remain childless, even if they undergo up to six cycles (Malizia *et al*., 2009). Subfertile couples should, therefore, be well informed about the chances of success with IVF/ICSI cycles before starting their first or before continuing with a new treatment (van Loendersloot *et al*., 2014). It is important to give the couple a real and fair expectation about their odds to get a live birth child, weighed against the risks of the treatment. On the other hand, since IVF/ICSI is expensive, the couple can decide if the financial (and emotional) burden can be justified (Hamdine *et al*., 2015). The threshold at which the couple will start or continue treatment may differ according to insurance company’s support, the taxpayers’ funds, and the patients own option (van Loendersloot et al., 2014). This probability of success is also important in the management of public Fertility Clinics and in the management of their waiting lists on public health national systems. To facilitate patient counseling, clinical decision-making, and access to health care provision, prediction models for live birth after IVF have been constructed (Nelson & Lawlor, 2011).

Fertility prediction models, before treatment, are treatment-independent and couples take part in these models before starting treatment (Zarinara *et al*., 2016). They can be based on patient baseline characteristics. Alternative models have incorporated the characteristics of the intermediate results of the first treatment cycle, thereby improving the accuracy of probability estimates for future cycles (La Marca *et al*., 2011).

A number of factors have been reported as influencing the success of IVF, either positively or negatively. Women’s age, antimullerian hormone (AMH) levels and antral follicle count (AFC) have been consistently shown to be associated with IVF success (Khader *et al*., 2013). During the last few years, some important pretreatment predictors, which used live birth as the primary outcome, have been published, and hereby we highlight them:

A - Nelson & Lawlor (2011). This predictor model was made using a cohort of 144,018 IVF cycles (data from Human Fertilization and Embryology Authority - HFEA) undertaken in the United Kingdom (UK) between 2003 and 2007, to examine the predictors of live birth with IVF treatment. This predictor includes woman’s age, infertility duration, source of eggs, cause of infertility, number of previous IVF cycles, previous pregnancies, medication use and type of treatment (IVF or ICSI). This model was built on considerably old datasets, pre-dating significant changes in clinical practice that occurred from 2008 onwards, therefore requiring a time adjustment. It was externally validated in 2014 (te Velde *et al*., 2014) and 2015 (Smith *et al*., 2015) , where it was found that it overestimated success rates. This predictor was used to produce a web calculator tool: www.ivfpredict.com.

B - La Marca *et al.* (2011). Unlike the previous model, which included a lot of predictors, La Marca and colleagues only included woman’s age and AMH in their model, as only these two factors were identified as predictive with the multivariate analysis done. They developed this predictor in an Italian cohort of 389 women, between 2005 and 2009 and demonstrated that AMH is significantly associated with live birth and that this association is independent of age. The external validation of Nelson’s predictor model, made by Khader *et al*. (2013), with 822 women in Glasgow, confirmed that AMH is an independent predictor of live birth, and AMH and age can be displayed as categories, rather continuous variables, with a clear benefit for applying the model in a clinical environment.

C - McLernon *et al*. (2016). For the model development, data from 113,873 women and 184,269 completed cycles (data from HFEA of UK) between 1999 and 2009 were used. This model is the first to provide an individualized estimate of the cumulative chance of a live birth over multiple complete cycles of IVF/ICSI, with a complete cycle defined as all fresh and frozen thawed embryo transfers resulting from one episode of ovarian stimulation cycle. In addition, it provides for a pre and posttreatment model. On the pretreatment model, the predictors included are woman’s age; duration of infertility, previous pregnancy; cause of infertility and type of treatment, not including AMH or AFC, ethnicity, BMI, smoking status or alcohol intake, since they were unavailable in the HFEA database. Internal validation of the model showed promising results, and they provided a web calculator https://w3.abdn.ac.uk/clsm/opis/tool/ivf1. Leijdekkers *et al*. (2018) recently validated this model in an independent prospective cohort of 1515 Dutch women who participated in the OPTIMIST trial, and underwent their ﬁrst IVF treatment between 2011 and 2014, in a total of 2881 completed cycles. They concluded that after minor recalibration of the pretreatment model, it proved valid in predicting the cumulative chance of a live birth after multiple complete treatment cycles in another geographical context, and that adding AMH, AFC and BMI data, it gained only a marginal improvement of the predictive performance (Leijdekkers *et al*., 2018). This validation can be questioned since it uses patients from a trial, who had done a restricted protocol with restricted doses, which in fact can lead to a different outcome, and they did not include anovulatory women, so it did not fully represent the overall patient population undergoing IVF/ICSI treatment.

D - Dhillon *et al*. (2016). In this predictor model, the authors intended to incorporate key pretreatment predictors, such as BMI, ethnicity and ovarian reserve. In this cohort study, a model to predict live birth was derived using data collected from 9915 women, who underwent IVF/ICSI treatment at any CARE (Center for Assisted Reproduction) clinic in the UK from 2008 to 2012. External validation was performed on data collected from 2,723 women who underwent treatment in 2013, which means, a different population in a different time, but at the same geographical place. The predictors that showed to have a significant effect on the chances of live birth were: age, tubal factor, unexplained causes of infertility and being south Asian or black, differently from other predictor models.

The predictor models already published do not consider all the possible pretreatment variables, even before figuring out if they are statistically significant or not. On the other hand, they are often not user-friendly on the patient perspective, or they reflect a reality different from the center where they will be used. Yet, they do not even explain in a simple way the couples’ odds have, although some of them have web calculators available.

In the attempt to provide more specific information to patients, and better fit our reality, our main goal is to design a simple predictor model, easily understandable.

## MATERIALS AND METHODS

A retrospective cohort study of all IVF/ICSI cycles started in our center between 2012-2016. Only cycles with a live birth delivery after 24 weeks, or cycles with no surplus embryos left were considered. Women’s age at oocyte retrieval varied between 18-39 years old. The following data was evaluated: AMH; AFC; women’s and men’s age; body mass index (BMI) both for men and women; smoking status; previous diagnosis; type of treatment (IVF/ICSI); having had previous deliveries. Since our model aims at pretreatment counseling only, we did not include any oocyte or embryo factors.

### IVF/ICSI procedures

According to local protocol, ovarian stimulation was performed with 100 to 450 IU of r-FSH or hMG (Gonal-f^®^, Merck Serono; Puregon^®^, MSD; Bemfola^®^, Gedeon Richter) or HMG (Menopur^®^, Ferring), based on ovarian reserve assessment, starting on cycle day 2 or 3, mostly within a GnRH antagonist flexible protocol (Cetrotide^®^, Merck Serono; or Orgalutran^®^, MSD) started on stimulation day 6. Final oocyte maturation was induced with hCG (mostly 6,500 IU Ovitrelle^®^, Merck Serono) or GnRH agonist (0.2 mg Decapeptyl^®^, Ferring) when at least two follicles of 17 mm in diameter were visualized by ultrasound. Oocyte retrieval was performed 35-37 hours after final maturation. ICSI was performed in cases of altered semen parameters, according to the World Health Organization (WHO) criteria or in cases of previous conventional IVF fertilization failure, or low fertilization rate. One or two embryos were transferred 2, 3 or 5 days after oocyte retrieval. A fresh transfer was canceled whenever the progesterone level was over 1,5 ng/mL, more than 18 oocytes were expected or intracavitary uterine pathology was identified during stimulation. The luteal phase was supplemented with vaginal micronized natural progesterone (200mg Progeffik^®^, Effik, three times a day). Supernumerary embryos of sufficient quality were cryopreserved on days 2, 3 or at blastocyst stage. Patients who did not become pregnant after fresh transfer could undergo frozen-thawed cycles under artificial endometrial preparation

Biochemical pregnancy was defined as a ß-HCG level>10 UI/L, 14 days after oocyte retrieval, and live birth was defined as at least one infant born alive after 24 weeks gestation, consistent with previous prediction models and publications. The hormonal measure of antimullerian hormone was done with blood serum sample using the Electrochemiluminescence (ECLIA) methodology, with the Modular EVO (E170) Roche Diagnostics® equipment.

### Statistical analysis

Univariate and multivariate analyses were used to examine the association of live birth with baseline patient characteristics. The odds-ratios were determined for all the statistically significant variables (*p*<0.05). The discrete variables were compared using the Chi-square test and the continuous ones with the t-student test. When necessary, the continuous variables were categorized. The results are presented according to the predictors founded. We used the SPSS 22.1 IBM software.

### Ethics

The Ethics Committee of our hospital approved this study.

## RESULTS

We evaluated 739 cycles. Cycle results and baseline characteristics of patients are described in Tables 1 and 2. Overall, 232 cycles ended up with at least one live birth. Of the 739 started cycles, 9.1% were canceled (without oocyte pick up); 1.1% did not have oocytes (n=7); 4% had no embryos (n=31) and 1.4% had no embryos for transfer because of poor quality (n=10). Almost half of the initiated cycles, 46%, had a β-HCG serum test positive and 31.4% of the cycles achieved a live birth. Overall, of the 624 cycles with day 2 embryos, 37% achieved a live birth (Table 1).

**Table 1:** Cycle’s results.

Initiated cycles	n=739
Without oocyte pick up (%)	67 (9.1%)
With pick, but without embryos for transfer (%)	48 (6.5%)
β-HCG +, n (%)	340 (46%)
Live birth per cycle started, n (%)	232 (31.4%)

### Univariate analysis

Concerning the continuous variables, there were no differences in the women’s age, AMH, AFC, women’s BMI, duration of infertility, men’s age and their BMI, and among the women who achieved a live birth and the ones who didn’t. Demographic factors such as ethnicity, smoking habits or previous children in both women and men were not statistically significant either (Table 2).

**Table 2:** Baseline characteristics of couples and their treatments

	Women with live birth n=232	Women without live birth n=507	%women with live birth	*p*-value
**Women's age (years), mean (SD)**	33.1 (3.8)	34.5 (3.6)		NS
**Women's age (years), sub-classified**				
** < 35, n (%)**	141 (60.8%)	230 (45.4%)	38%	<0.000
** 35-37, n (%)**	65 (28%)	159 (31.4%)	29%	
** 38-39, n (%)**	26 (11.2%)	118 (23.3%)	18.1%	
**Exponential age**	1,65234 E+16	8,05341 E+15		<0.000
**Women's BMI (kg/m^2^), mean (SD)**	24.2 (4.2)	24.5 (5.4)		NS
**Women’s smoking status, n (%)**				
** Present**	66 (28.4%)	134 (26.4%)	33%	NS
** Previous**	36 (15.5%)	82 (16.2%)	30.5%	
** Never**	130 (56%)	291 (57.4%)	30.9%	
**Women’s previous children. n (%)**				
** previous**	20 (8.6%)	53 (10.4%)	31.8%	NS
** No previous children**	212 (91.4%)	454 (89.5%)	27.4%	
**Women’s ethnicity. n (%)**				
** Caucasian**	208 (89.7%)	454 (89.5%)	31.4%	NS
** African**	6 (2.6%)	23 (4.6%)	20.7%	
** Asiatic**	1 (0.4%)	2 (0.4%)	33.3%	
** Gipsy **	5 (2.2%)	6 (1.2%)	45.5%	
** Indian**	2 (0.9%)	3 (0.6%)	40%	
** Mixture**	10 (4.3%)	19 (3.7%)	34.5%	
**Infertility duration (months), mean (SD)**	55.2 (29.4)	57.0 (28.4)		NS
**Men’s age, mean (SD) **	35.4 (4.9)	36.5 (5.3)		NS
**Men's BMI (kg/m^2^), mean (SD) **	26.7 (11.7)	26.3 (3.9)		NS
**Men’s smoking status, n (%)**				
** Present**	82 (35.3%)	161 (31.8%)	33.7%	NS
** Previous**	33 (14.2%)	91 (17.9%)	26.6%	
** Never**	117 (50.4%)	255 (50.3%)	31.5%	
**Men’s previous children, n (%)**				
** Previous children**	25 (10.8%)	68 (13.4%)	26.9%	NS
** No previous children**	207 (89.2%)	439 (86.6%	32%	
**Men’s ethnicity, n (%)**				
** Caucasian**	203 (87.5%)	475 (93.7%)	29.9%	NS
** African**	8 (3.4%)	13 (2.6%)	38.1%	
** Asiatic**	1 (0.4%)	2 (0.4%)	33.3%	
** Gipsy **	6 (2.6%)	6 (1.2%)	50%	
** Indian**	3 (1.3%)	3 (0.6%)	50%	
** Mixture**	11 (4.8%)	8 (1.6%)	57.9%	
**AFC, mean (SD) **	17.0 (9.2)	13.0 (9.0)		NS
**Logarithmized AFC **	2.698	2.379		<0.000
**AFC sub-classified**				
** AFC 0-6 **	15 (6.5%)	111 (21.9%)	11.9%	<0.000
** AFC 7-10**	44 (18.9%)	145 (28.6%)	23.3%	
** AFC >10**	173 (74.6%)	251 (49.5%)	40.8%	
**AMH (ng/mL), mean (SD)**	4.2 (3.8)	3.2 (3.7)		NS
**Logarithmized AMH **	1.108	0.629		<0.000
**AMH sub-classified**				
** <0.7, n (%) **	9 (3.9%)	55 (11.8%)	9.6%	<0.000
** 0.7-1.19, n (%)**	16 (6.9%)	60 (10.8%)	18.8%	
** >1.2, n (%)**	207 (89.2%)	324 (77.4%)	37%	
**Infertility cause**				0.482
** Endometriosis**	19 (8.2%)	49 (9.7%)	27.9%	
** Ovulatory**	21 (9.1%)	36 (7.1%)	36.8%	
** Tubal**	33 (14.2%)	81 (16.0%)	28.9%	
** Male factor**	86 (37.1%)	151 (29.8%)	36.3%	
** Mix of female factors**	6 (2.6%)	17 (3.4%)	26.1%	
** Mix of male and female factors**	16 (6.9%)	51 (10.1%)	23.9%	
** Unexplained**	49 (21.1%)	118 (23.3%)	29.3%	
** Others**	2 (0.9%)	4 (0.8%)	33.3%	
**Infertility cause (grouped) **				
** Ovulatory or Male Factor**	107 (46.1%)	187 (36.7%)	36.4%	0.017
**Any other infertility cause**	125 (53.9%)	320 (63.1%)		
**Type of fecundation, n (%) **				NS
** IVF**	122 (52.6%)	251 (57.2%)	32.7%	
** ICSI**	110 (47.4%)	188 (42.8%)	36.9%	

AMH – Antimullerian hormone; AFC – antral follicle count; BMI – Body mass index.

As age, AFC and AMH have been commonly associated with live birth rates, these variables were transformed in two different ways: age was exponentialized and AMH and AFC were logarithmized, as these curves better describe the expected behavior of these variables on reproductive outcomes after IVF/ICSI. On the other hand, these variables were categorized in three classes to design the patient-friendly final model. In both transformations, these variables were highly statistically significant (*p*<0.001) (Table 2). In a post-hoc sub-group analysis, we noticed that couples undergoing treatment for ovulation disorder or pure male factor seemed to have a more favorable scenario. When we tested this group against all other causes, we noticed that this was also statistically significant (*p*=0.017).

### Multivariate analysis

For the multivariate analyses, we performed a binary regression. Only the categorized women’s age, AFC, AMH and male factor or ovulatory factor showed to be statistically significant to achieve a live birth. The p-value for the Hosmer and Lemeshow test was 0,740. The ROC curve had an area under the curve of 0.688 (IC 0.649-0.728) (Table 3). The data from the regression results are simplified in Table 4.

**Table 3:** Regression results

	*p*-value	OR	Odds ratio
**AMH**			
<0.7	0.1	0.355	-2.81
0.7-1.2	0.01	0.598	-1.67
>1.2	0.018		REFERENCE
**AFC**			
0-6	0.004	0.364	-2.7
7-10	0.003	0.545	-1.8
>10	0.001		REFERENCE
**Age**			
<35	0.002	2.153	2.15
35-37	0.041	1.74	1.75
38-39	0.009		REFERENCE
**Male factor or ovulatory**	0.029	1.447	+1.5

**Table 4:** Model probabilities according to age, AMH, AFC and cause of infertility

		< 35 years			35-37 years			38-39 years		
	AMH	<0.7	0.7-1.19	>1.2	<0.7	0.7-1.19	>1.2	<0.7	0.7-1.19	>1.2
	AFC									
**Other causes**	**0-6**	8.5%	13.6%	20.8%	7.0%	11.3%	17.5%	5.9%	6.8%	10.9%
	**7-10**	12.3%	19.1%	28.3%	10.2%	16.0%	24.2%	8.6%	9.9%	15.5%
	**> 10**	20.4%	30.2%	42.0%	17.2%	26.0%	36.9%	10.7%	16.7%	25.2%
**Male or ovulatory factor**	**0-6**	11.9%	18.6%	27.6%	9.8%	15.6%	23.5%	4.2%	9.6%	15.0%
	**7-10**	16.8%	25.5%	36.3%	10.2%	21.6%	31.6%	6.1%	13.7%	20.9%
	**> 10**	27.1%	38.5%	51.1%	23.1%	33.6%	45.8%	14.7%	22.5%	32.7%

## DISCUSSION

The IVF/ICSI treatment predictors can consider pre and post-treatment variables. The pretreatment models estimate the probability of a live birth using the characteristics of the couple when they intend to undergo an IVF/ICSI cycle, such as the woman’s age, duration of infertility, type of infertility, previous pregnancy status of the couple, ovarian reserve and/or its biomarkers, and treatment type. The post-treatment variables include treatment-specific characteristics (number of oocytes, cryopreservation of embryos, and the number and stage of embryos) from the complete cycles, along with the characteristics of the couple from the pretreatment model, in order to update the cumulative probability of achieving a live birth (Leijdekkers *et al*., 2018; McLernon *et al*., 2016).

Yet, some models predict the probability of a live birth after a single fresh embryo transfer only, excluding the important contribution of embryo cryopreservation and subsequent treatment cycles to cumulative live birth rates (Leijdekkers *et al*., 2018). They failed to consider all embryo transfer attempts, which means that such prediction models are not useful as counseling tools, underestimating the odds of success.

Regarding the woman’s age, considered one of the most important predictors, it seems logical to include it in the prediction models (Leijdekkers *et al*., 2018; Khader *et al*., 2013), which, on the other hand, does not occur with AMH. Hamdine *et al*. (2015) demonstrated that although AMH added some value in predicting the 1-year cumulative live birth rate, its predictive accuracy was limited and added little to prognosis based on the female age alone. The model published by McLernon *et al*. (2016) and its external validation by Leijdekkers *et al*. (2018) did not include AMH. The last ones consider that the addition of ovarian reserve measures, i.e. AMH and AFC, to the prediction models revealed only a marginal improvement, stressing the extra costs and physical burden on the patient (Leijdekkers *et al*., 2018; Broer *et al*., 2013). However, in one large study (Nelson *et al*., 2007), AMH was shown to be associated with live births, regardless of age after treatment, and recently a further large cohort study demonstrated that serum AMH concentrations may predict live births in women older than 34 years of age (Lee *et al*., 2009). Other potential predictors for live birth, such as ethnicity, smoking habit and alcohol intake, can be considered. The additional value of these variables for model performance are considered uncertain, as the reporting is remarkably subjective and/or often incomplete (Leijdekkers *et al*., 2018; McLernon *et al*., 2016).

In our study, when we used age, AMH and AFC, as continuous variables, there is no statistical differences among the groups. However, since the relation of these variables with the outcome is not linear in the literature, we decided to transform these variables in two different ways. On the one hand, we categorized them and, on the other hand, we exponentialized age and logarithmized AMH and AFC. With both modeling we had a highly statistically significant difference (*p*<0.001). Despite the controversial data on ovarian reserve measures and their importance in prediction models, in our study they actually showed an important relation with the outcome, live birth, improving the accuracy and making this predictor more reliable and user-friendly to patients. After a post hoc analysis, we also noticed that the couples undergoing treatment for ovulation disorder or pure male factor had an odds ratio of 1.5 for the outcome and, therefore, we decided to include it in the final model.

In Portugal, the last published results are from 2015 (CNPMA, 2017). There, the overall cumulative delivery rate varies between 25-30% (FIV/ICSI) per started cycle. Our model has the advantage of reflecting our particular population and our particular work setting. On the other hand, it can be simplified in a small table and it had a good overall result in the ROC curve (0.688), especially when compared with other models.

This model has some limitations. One of them was that it did not consider the couples’ previous treatments. In fact, some patients had done previous treatments in other clinics and we could not access their data. Another limitation is that sub-groups were created after a post hoc analysis of the data and this might be a source of bias. The consistency of these differences should be confirmed in other studies. We are now planning to validate this model prospectively, first in our population and then in other clinical settings.

## CONCLUSION

Age, AMH and AFC, when sub-classified, are independently associated to the results of an IVF/ICSI treatment. The cause of infertility was also importantly associated when sub-categorized as male or ovulatory factor vs others. It is possible to calculate the final treatment results based on a predictor. This predictor is easily understandable and can work as an important tool to help counseling patients in a daily basis. It can grade patients’ probability of success in achieving a live birth between 5.9% and 51.1%.
